# Hybrid Molecules Consisting of Lysine Dendrons with Several Hydrophobic Tails: A SCF Study of Self-Assembling

**DOI:** 10.3390/ijms24032078

**Published:** 2023-01-20

**Authors:** Oleg V. Shavykin, Sofia E. Mikhtaniuk, Emil I. Fatullaev, Igor M. Neelov, Frans A. M. Leermakers, Mariano E. Brito, Christian Holm, Oleg V. Borisov, Anatoly A. Darinskii

**Affiliations:** 1 School of Computer Technologies and Control, St. Petersburg National Research University of Information Technologies, Mechanics and Optics (ITMO University), Kronverkskiy pr. 49, 197101 St. Petersburg, Russia; 2 Physics Department, Lomonosov Moscow State University, Leninskie Gory 1-2, 119991 Moscow, Russia; 3 Department of Mathematics, Tver State University, Sadoviy per., 35, 170102 Tver, Russia; 4 Physical Chemistry and Soft Matter, Wageningen University, 6703 NB Wageningen, The Netherlands; 5 Institute for Computational Physics, University of Stuttgart, D-70569 Stuttgart, Germany; 6 Institute of Macromolecular Compounds of the Russian Academy of Sciences, 199004 St. Petersburg, Russia; 7 Institutdes Sciences Analytiques et de Physico-Chimie pour l’Environnementetles Matériaux, UMR 5254 CNRS UPPA, 64053 Pau, France

**Keywords:** drug delivery systems, peptide dendrimer, self-assembly, computer simulation, self consistent field, electrical double layer

## Abstract

In this article, we used the numerical self-consistent field method of Scheutjens–Fleer to study the micellization of hybrid molecules consisting of one polylysine dendron with charged end groups and several linear hydrophobic tails attached to its root. The main attention was paid to spherical micelles and the determination of the range of parameters at which they can appear. A relationship has been established between the size and internal structure of the resulting spherical micelles and the length and number of hydrophobic tails, as well as the number of dendron generations. It is shown that the splitting of the same number of hydrophobic monomers from one long tail into several short tails leads to a decrease in the aggregation number and, accordingly, the number of terminal charges in micelles. At the same time, it was shown that the surface area per dendron does not depend on the number of hydrophobic monomers or tails in the hybrid molecule. The relationship between the structure of hybrid molecules and the electrostatic properties of the resulting micelles has also been studied. It is found that the charge distribution in the corona depends on the number of dendron generations *G* in the hybrid molecule. For a small number of generations (up to G=3), a standard double electric layer is observed. For a larger number of generations (G=4), the charges of dendrons in the corona are divided into two populations: in the first population, the charges are in the spherical layer near the boundary between the micelle core and shell, and in the second population, the charges are near the periphery of the spherical shell. As a result, a part of the counterions is localized in the wide region between them. These results are of potential interest for the use of spherical dendromicelles as nanocontainers for drug delivery.

## 1. Introduction

The self-assembly of molecules consisting of hydrophobic and hydrophilic parts in water solution is a well-known effect attracting the attention of researchers for many years. The variation of the architecture and chemical structure of both parts produces a large number of aggregates of different sizes and morphologies (spherical, cylindrical, or lamellar).

Self-organizing structures composed of amphiphilic molecules are important because they exist in all living organisms. Their structures are also widely used in many industrial applications, such as, lubrication, wetting, cleaning, formulation sciences, and nanotechnology [1,2,3]. Micelles consisting of linear amphiphilic diblock copolymers were used for a very long time as nanocontainers in drug and gene delivery. Dendromicelles consisting of hybrid molecules containing hydrophilic dendron block and hydrophobic linear blocks are a perfect candidate for use in this area [4,5,6,7]. The large number of hydrophilic or charged terminal groups of dendron on surface of micelle expands the scope of possible applications of such systems. However, not all dendromicelles are suitable for biomedical applications because they should have low toxicity and high biocompatibility. From this point of view, peptide, and in particular lysine dendrons, are among the most suitable candidates for use in this area. Lysine dendrons were synthesized in the early 80s [8]. Despite a large number of works devoted to the applications of lysine dendrimers, the relationship between the structure of these hybrid molecules and the characteristics of the resulting dendromicelles remains insufficiently studied. Several papers describe the synthesis of such molecules consisting of one lysine dendron with one [9], two, and three [10] hydrophobic tails. The detailed experimental study of the self-assembly of such hybrid molecules was performed only for molecules consisting of one lysine dendron (generation *G* = 0–2) and one hydrophobic alkyl tail of different lengths (from C4 to C18) by using small angle neutron scattering (SANS) and dynamic light scattering (DLS) [9].

Synthesis of linear-dendritic amphiphilic block-copolymers with many linear hydrophobic tails was described in [11,12,13,14,15,16] and responsive assemblies of such molecules were studied in [17,18,19,20,21,22,23]. General applications of them in biotechnology are described in [24,25,26,27]. Bio-, photo- and pH-degradable linear-dendritic block-copolymers systems were studied in [28,29] and applications of these molecules in drug and gene delivery were described in [30,31,32,33,34]. The synthesis of more complex amphiphilic Janus-dendrimers consisting of two branched block was described in [35]; the study of dendrisomes based on Janus-dendrimers and their applications in drug delivery were described in [36,37,38,39,40,41,42].

In this paper, we study the spherical micelles based on linear-dendritic amphiphilic block-copolymers with many linear hydrophobic tails because they are more convenient for application in drug and gene delivery. Our first goal is to determine the values for structural parameters of the hybrid molecule (the generation number of dendron blocks (*G*), the number of tails (*t*), and the tail length ($N_{t}$)), where the formation of micelles of spherical morphology is expected. The second goal is to establish the relationships between the size and inner structure of these micelles and the parameters of a hybrid molecule. Previous works [43,44,45] allow us to make some general predictions about the connection between the structure of polymeric surfactants and resulting aggregates. These works consider mainly micelles formed by macromolecules containing only linear hydrophilic and hydrophobic blocks. Only recently, a theory applicable on micelles formed by linear-dendritic block copolymers was developed [46]. It was shown that an increase in the number of dendron generations leads to a decrease in the aggregation number.

However, in general, the theory predicts only trends and does not take into account the specific chemical structure of the systems under consideration. In addition, the theory is developed for neutral dendrons, while lysine dendrons remain charged even under physiological conditions. This specificity can be considered by using computer simulations [47]. The most accurate method of simulation of large molecular systems is molecular dynamics (MD) [47,48,49]. However, it takes considerable computational resources for long term simulation of big systems such as micelles consisting of many linear-dendron molecules in explicit solvent. In addition, when applying the MD method to study self-organization, a problem of using the grand canonical ensemble arises. Namely, it is necessary to consider the whole ensemble of systems, which further increases the required amount of MD calculations. Brownian dynamics [47] and dissipative particle dynamics [47,50,51,52] methods are faster in comparison with full-atom MD, since these methods usually use more coarse-grained models and implicit solvents. However, both BD and DPD calculations could be, also, too expensive due to the slow equilibration of weakly interacting micelles.

The much less time-consuming numerical self-consistent field (SCF) approach can be positioned as a transitional method between computer simulation and analytical theory. This method is based on the thermodynamics of small systems and allows the determination of the equilibrium characteristics of the most probable micelles [53,54,55,56,57,58,59,60,61,62,63,64]. In our previous work, we used this method to study the self-assembly of hybrid molecules consisting of lysine dendrons with one hydrophobic tail in water solution. The relationship between parameters of hybrid molecule (the number of dendron generations and the length of tail) and the size and structure of resulting micelles was established. The calculation results were in satisfactory agreement with the available experimental data.

In the present work, we apply the SCF method to study the self-assembly of more complex hybrid molecules containing lysine dendron and several hydrophobic tails attached to its root. The outline of the paper is as follows. In Section 3, the models and the method are described. Section 2 contains the results of the calculations. Section 4 concludes the paper.

## 2. Results and Discussion

This section has the following organization. In the beginning, the boundaries between different micelle morphologies—sphere-cylinder and cylinder-lamellae—will be presented in the space of parameters: generation number *G* of the hydrophilic head and the length of the hydrophobic tail. The areas of the existence of spherical micelles will be identified for molecules with various numbers of tails: t=1, 2, 4, and 6 (for each molecule, all tails have the same length). All calculations (unless otherwise stated) were performed at a dimensionless volume concentration of the salt equal to 0.01. This corresponds to the salt concentration of 0.1M used in the experimental work [9].

### 2.1. Thermodynamic Characteristics

**Figure 1:**
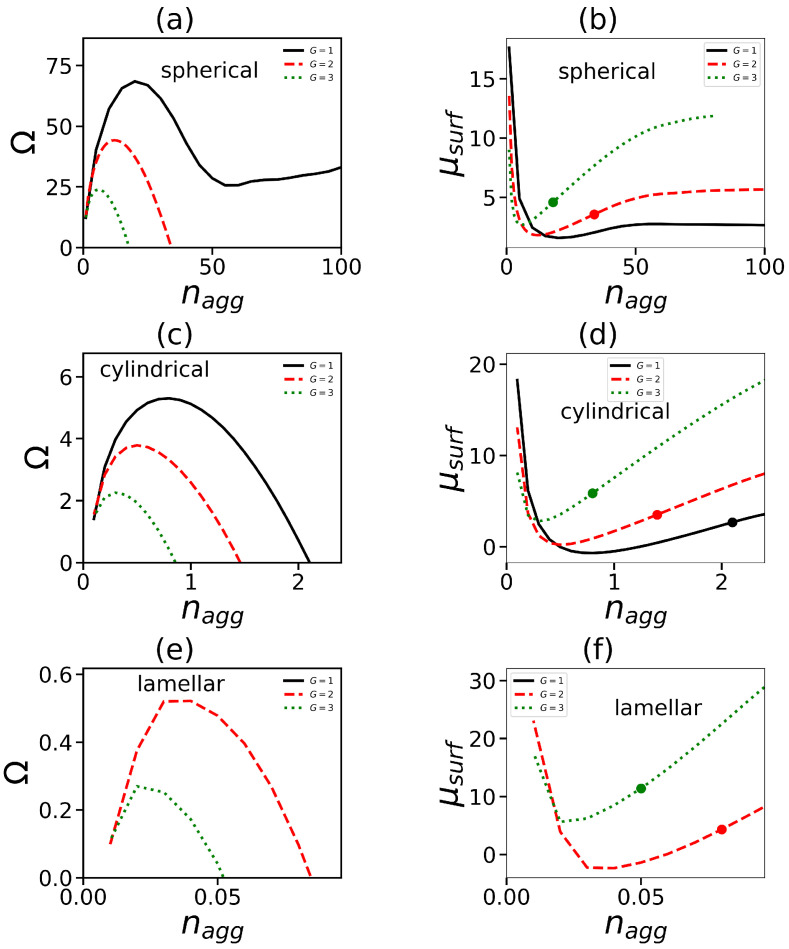
The grand potential Ω (**a**,**c**,**e**) and the chemical potential $\mu_{surf}$ (**b**,**d**,**f**) for spherical (**a**,**b**), cylindrical (**c**,**d**), and lamellar (**e**,**f**) geometries as functions of the aggregation number $n_{agg}$. Model of surfactant with t=4 tails and the length of one tail $N_{t}=15$.

We will determine the boundaries between different micelles morphologies by using the dependencies of the Grand potential $\Omega (n_{agg})$ and chemical potential $\mu_{surf}$ on the aggregation number $n_{agg}$. Recall that $\Omega (n_{agg})$ is equal to the work of the formation of most probable micelles with aggregation number equal to $n_{agg}$. A detailed description of the methodology for calculation of the potential $\Omega (n_{agg})$ in the framework of the SF-SCF method is given in [65]. According to this approach, Ω does not include the entropic term connected with the translational degrees of freedom. Figure 1 demonstrates the typical behavior of the Grand thermodynamic potential $\Omega (n_{agg})$. It shows, as an example, the dependencies $\Omega (n_{agg})$ for molecules with one hydrophilic dendron head (the generation numbers G=1,2,3) and four tails. Each tail has the same length, $N_{t}=15$. Calculations were performed for spherical, cylindrical, and lamellar forms of the aggregate.

At first, it grows with $n_{agg}$ (the region of the pre-micellar aggregates), then passes through the maximum (which corresponds to the critical concentration of the micelle formation). At the further increase in $n_{agg}$, the potential Ω decreases because adding surfactant molecules to the aggregate reduces its surface tension. In most cases, Ω drops to zero. The value of $n_{agg}$ corresponding to Ω=0 is considered as the aggregation number of a most probable micelle with given morphology. If Ω does not reach zero, a micelle of this morphology (spherical in Figure 1a) is not formed for this surfactant structure. It should be noted that such behavior of Ω is observed at not-too-high concentrations of surfactants in solution. At high surfactant concentrations, Ω can drop below zero. This is due to the fact that the dilute solution condition for micelles and, thus, the assumption of the existence of non-interacting micelles are not fulfilled. In our case, we consider low concentrations where the dilution criterion is met.

The next step is to select the most probable morphology of the micelle for a given surfactant. For each of these morphologies, we find the chemical potential $\mu_{surf}$ of the surfactant molecule under the condition of complete equilibrium. Chemical potentials $\mu_{surf}$ cannot depend on a spatial coordinate when they are in equilibrium. That is why we can evaluate it using bulk concentrations only. Hence, results are similar to the ones that follow from the Flory-Huggins theory

(1) $$\begin{array}{c} \mu_{surf}=-\ln\varphi_{surf}^{b}+1-N_{surf}\sum_{j}\frac{\varphi_{j}^{b}}{N_{j}}-\\ \frac{N_{surf}}{2}\sum_{X}\sum_{Y}\left(\varphi_{X}-\frac{N_{surf,X}}{N_{surf}}\right)\chi_{XY}\left(\varphi_{Y}^{b}-\frac{N_{surf,Y}}{N_{surf}}\right) \end{array}$$

wherein $N_{surf}$ is the total number of surfactants in the system, $N_{surf,Y}$ is the number of segments *Y* in surfactant. The physical sence of the chemical potential is the work required to add one more surfactant molecule to an aggregate of a given morphology.

The values of $\mu_{surf}$ were calculated by using Formulae (1). According to the formula

(2) $$\Omega =F-\mu_{surf}n_{agg}$$

Ω=0 corresponds to the equation $F=\mu_{surf}\cdot n_{agg}$. As mentioned above, we neglect the contribution to free energy corresponding to the translational entropy of the micelle. Therefore, the micelle morphology with a lower chemical potential is considered the most favorable. The point where the chemical potentials of different morphologies are equal will correspond to the transition point between them. Figure 1b,d,f show the dependencies of the chemical potential $\mu_{surf}$ on the aggregation number corresponding to different micelle morphologies.

**Figure 2:**
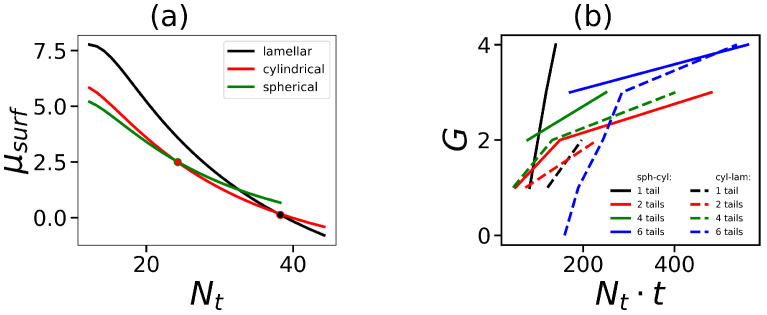
(**a**) Dependence of the chemical potential on the length of one hydrophobic tail (the overall number of tails t=4). The lower the chemical potential, the more favorable that morphology. The dots on the curves show points of transitions between different morphologies (sphere-cylinder, cylinder-lamellar). (**b**) Diagrams of states of the micelle in coordinates of the overall number of segments in hydrophobic tails in the surfactant molecule $N_{t}\cdot t$ and the generation number *G* in the hydrophilic dendron. The regions of spherical micelles and lamellar structures are above the solid and below dashed lines, correspondingly. The intermediate region corresponds to the cylindrical configuration of micelles.

In Figure 2, the dependencies of the value of the chemical potential at Ω=0 on the length of the hydrophobic tail are illustrated for three different morphologies. The dots show points of the morphological transitions, i.e., when the chemical potential of one morphology becomes less than the other (i.e., one morphology becomes more favorable). It is seen that at small values of $N_{t}$, the spherical form is the most preferable, then there is a transition to the cylindrical form, and only at very long $N_{t}$ the lamellar morphology begins to dominate. The calculations were performed for different *G* and $N_{t}$, and points of transitions between spherical, cylindrical, and lamellar states were determined. Figure 2b demonstrates the boundaries between different morphologies. Since we are interested in spherical micelles, it is necessary to consider the boundary between spherical and cylindrical micelles. It is seen that at the same number of hydrophobic segments in the surfactant, the boundary shifts to higher *G* by the increase in the number of tails. The slope of the curves decreases as well. This means that in order to form spherical micelles, hybrid molecules with a small number of long tails must have dendron heads with a larger number of generations than molecules containing a large number of shorter tails.

**Figure 3:**
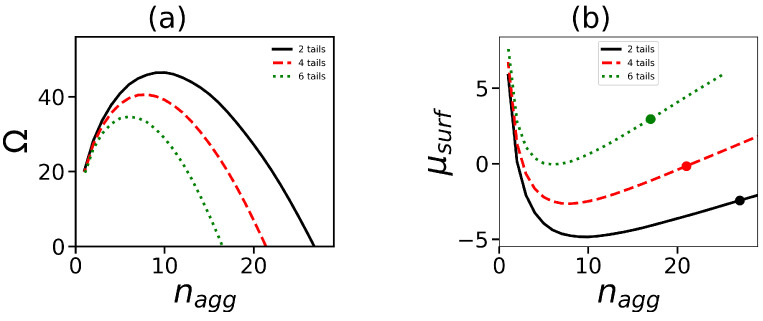
Dependencies of (**a**) the grand potential $\Omega (n_{agg})$ and (**b**) the chemical potential $\mu (n_{agg})$ at the same generation number G=4, and the same overall number of hydrophobic segments in the surfactant $N_{t}\cdot t=120$ (t=2, $N_{t}=60$; t=4, $N_{t}=30$; t=6, $N_{t}=20$). The dots in (**b**) show the values of $\mu_{surf}$ where $\Omega (n_{agg})=0$.

Let us now consider the effect of the number of tails on the aggregation number of spherical micelles. This effect can be estimated from the analysis of dependences of the Grand potential $\Omega (n_{agg})$ and chemical potential $\mu_{surf}\left(n_{agg}\right)$. Figure 3 shows an example of such dependences under a constant overall number of hydrophobic segments per one surfactant molecule. It is seen that the redistribution of hydrophobic segments over a larger number of tails leads to the formation of stable spherical micelles with smaller aggregation numbers. This result agrees qualitatively with the theoretical results of the work [46] where micelles formed by copolymers consisting of branched hydrophilic and hydrophobic blocks were considered. It was shown that the branching of the hydrophobic block at the same number of hydrophobic units decreases the aggregation number and size of the micelle.

**Figure 4:**
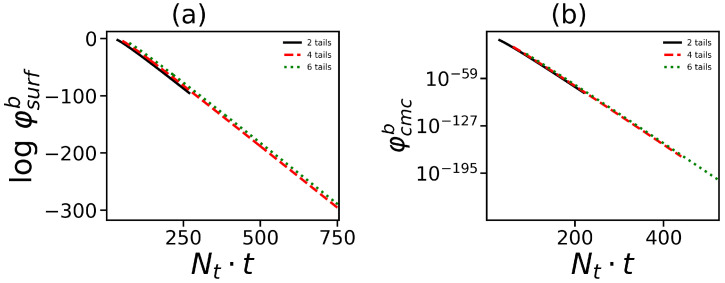
Dependence of (**a**) the volume fraction (dimensionless) $\varphi_{surf}^{b}$ of free surfactant molecules and (**b**) the critical micelle concentration $\varphi_{c.m.c.}^{b}$ on the overall number of monomeric units in all tails at different numbers of tails. Data for the same generation number G=4 are shown.

It is important to note that there is strong theoretical and practical interest in the effect of the number of tails on such important characteristics of the micelle solution as the volume fraction of surfactant molecules in the free volume $\varphi_{surf}^{b}$ as well as the critical micelle concentration (c.m.c.) $\varphi_{c.m.c.}^{b}$. Our calculations (see as an example Figure 4) show that these characteristics ($\varphi_{surf}^{b}$ and $\varphi_{c.m.c.}^{b}$) are insensitive to the number of tails and depend only on the overall number of hydrophobic segments in the hybrid molecule. Both of these values decrease with growth of $N_{t}\cdot t$. This is due to the fact that a larger number of the hydrophobic segments in the molecule results in a higher «driving» force of self-assembly, which means that self-assembly occurs at smaller surfactant concentration and the proportion of free surfactants becomes smaller.

### 2.2. Structure

**Figure 5:**
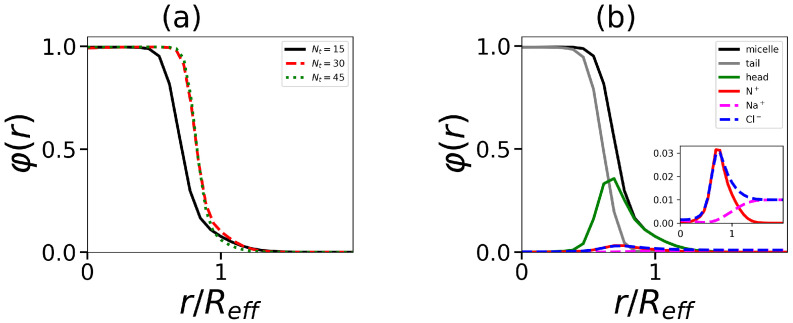
The normalized density profiles (by effective radius $R_{eff}$, which are defined below) (**a**) for segments in micelle with tail length $N_{t}=15,\;30,\;45$ and (**b**) the distribution of tail (grey), head groups (green), and overall (black) for hydrid molecule with tail length $N_{t}=15$. The distributions of the charged group in the dendron (red), co-ions, and counterions (pink and blue) are also given in the inset in an enlarged scale. All cases correspond to the same number of tails t=4 and generation number G=4.

The next part of our research is the study of the internal structure of the micelles and its dependence on the structure of hybrid molecules. One of the characteristics of the structure is the density profile φ(r) (the dependence of the local volume fraction of segments of different type in the spherical micelle on the distance *r* from the center of mass). An example of the density profiles for micelles formed by surfactants containing a dendron head with generation number G=4 and four tails of different length is shown in Figure 5.

Data are shown for all segments and separately for segments in dendron heads and in hydrophobic tails. Density profiles for charged groups in dendrons, counterions, and salt ions are displayed as well. The micelle consists of the dense core and the looser corona. With an increase in $N_{t}$, the boundary between core and corona becomes at first more sharp (see the transition from $N_{t}=15$ to $N_{t}=30$ in Figure 5a), and then its sharpness stabilizes (see the transition from $N_{t}=30$ to $N_{t}=45$). Figure 5b shows the distributions of charged groups and counterions for the micelle consisting of surfactant with the tail length $N_{t}=15$. It is seen that counterion distribution entirely overlaps with that of charged groups.

**Figure 6:**
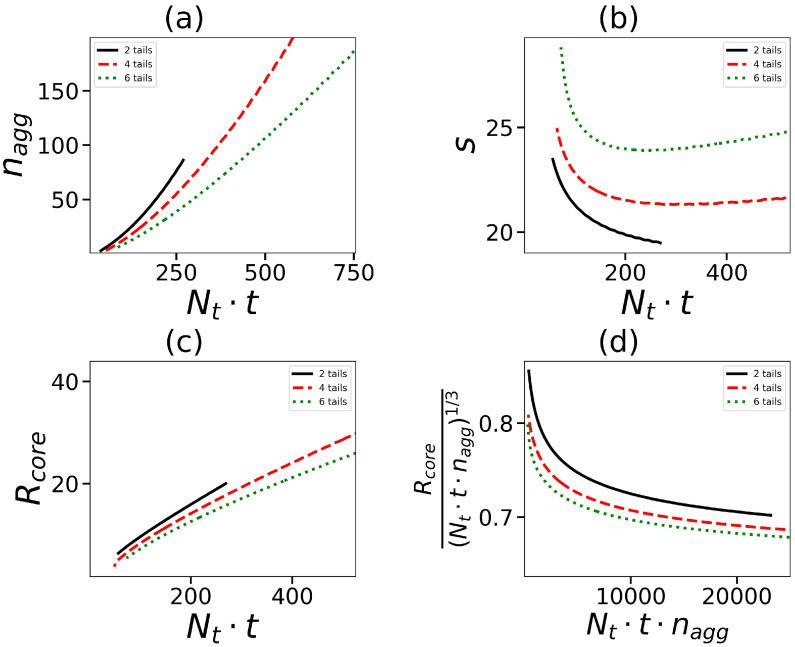
(**a**) The aggregation numbers $n_{agg}$, (**b**) the surface area *s* per one dendron of a core, (**c**) the size of micelle core $R_{core}$ as a function of the overall number of units in tails $N_{t}\cdot t$. (**d**) $R_{core}/{\left(N_{t}\cdot t\cdot n_{agg}\right)}^{1/3}$ as a function of the overall number of units in core $N_{t}\cdot t\cdot n_{agg}$. All cases correspond to the same generation number G=4.

Let us now consider how the total number of hydrophobic segments and the number of tails affect micelle characteristics such as the aggregation number $n_{agg}$ and core radius $R_{core}$. The last one was calculated as the second moment of the density distribution for hydrophobic segments. Figure 6a,c show that both these characteristics, as expected, increase with the number of hydrophobic segments in the hybrid molecule. However, the splitting of hydrophobic monomers over the larger number of tails leads to the decrease in these characteristics. These results are in agreement with data obtained in the previous section (see Figure 3).

The total number of hydrophobic segments in the core is equal to $N_{t}\cdot t\cdot n_{agg}$. The ratio $R_{core}/{\left(N_{t}\cdot t\cdot n_{agg}\right)}^{1/3}$ can be used for the characterization of the stacking density of hydrophobic segments in the core. Figure 6d shows the initial increase in this density at a small number of segments and the subsequent reaching of a practically constant value at a large $N_{t}\cdot t\cdot n_{agg}$. The splitting of segments over a larger number of tails leads to an increase in the core density at the same total number of hydrophobic segments in the micelle.

**Figure 7:**
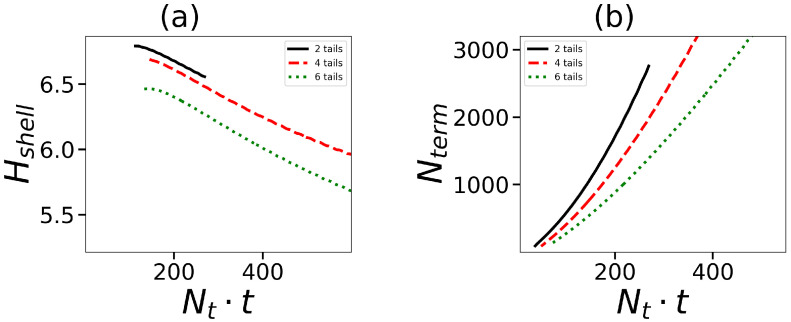
(**a**) The size of shell $H_{shell}$ of micelle corona (corona thickness) and (**b**) the number of terminal groups of dendrons in the micelle as a function of the overall number of units in tails $N_{t}\cdot t$. All cases correspond to the same generation number G=4.

Let us now turn to the characteristics of the micelle corona. The layer of dendrons forming the corona can be considered as a spherical brush with a grafting density of 1/s, where s is the area per dendron on the core surface. Figure 6b shows the dependence of *s* on the overall number of hydrophobic segments in the surfactant molecule. At first *s* decreases with $N_{t}\cdot t$ and then goes to a constant value. The greater the number of tails (at the same $N_{t}\cdot t$), the denser the dendrons are located on the surface of the core. It is well known that grafted dendrons in the brush are stretched due to the repulsion between neighboring chains. The stretching decreases with an increase in *s*, and $N_{t}\cdot t$. It should be expected that the thickness of the corona $H_{shell}$ decreases as well. Figure 7a confirms this conclusion.

The corona thickness $H_{shell}$ was determined as

(3) $$H_{shell}=R_{m}-R_{core}$$

where $R_{m}$ is the second moment of the distribution of terminal segments of dendrons in the micelle. It is seen that the behavior of $H_{shell}$ is opposite to that of $R_{core}$. At the same $N_{t}\cdot t$, the corona thickness, decreases with an increase in the number of tails.

Another characteristic of a micelle formed by hybrid molecules is the overall number $N_{term}=\int n_{t}\left(r\right)\cdot dr$ of terminal groups of dendrons in the micelle. This characteristic is essential, particularly for biomedical applications such as drug delivery, because these groups can be functionalized. The dependence of $N_{term}$ on the number and length of hydrophobic tails in the surfactants forming the micelle is demonstrated in Figure 7b. As an example, the data for spherical micelles with G=4 are shown. The value of $N_{term}$ naturally increases with an overall number of hydrophobic segments. The increase in the number of tails decreases $N_{term}$ because the aggregation number (and the number of dendrons) is larger for surfactants with a small number of tails (see Figure 6a).

### 2.3. Electrostatic Properties

**Figure 8:**
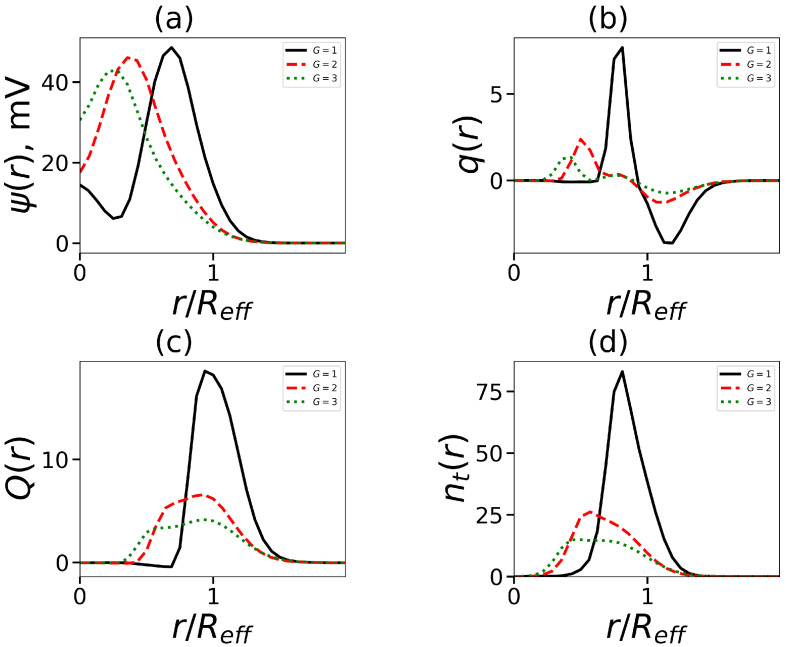
The normalized radial distribution (by effective radius $R_{eff}$, which is defined below) (**a**) the electrostatic potential ψ, (**b**) the dimensionless charge q(r), (**c**) the cumulative charge Q(r), and (**d**) the terminal charged groups of dendrons in the micelle. All cases correspond to the same number of tails t=2, tail length $N_{t}=15$.

As mentioned above, lysine dendrons have charged groups. Therefore, the question arises about the features of the electrostatic properties of micelle formed hybrid molecules with lysine heads. One of such characteristics is a distribution of the electrostatic potential ψ(r). Figure 8a shows these distributions for micelles formed by molecules with the same tail length and number of tails, but with a different number of dendron generations. All curves have a maximum that shifts to the core with an increase in *G*. This shift reflects the decrease in the aggregation number).

Another characteristic is the charge distribution q(r)

(4) $$q\left(r\right)=4\pi \left(\varphi_{term}\left(x\right)+\varphi_{Na}\left(r\right)-\varphi_{Cl}\left(r\right)\right)\cdot r^{2}$$

Figure 8b shows, as an example, q(r) for micelles formed by surfactants with two hydrophobic tails ($N_{t}=15$) and a dendron head of generation *G* = 1–3. These molecules form spherical micelles. For G=1, the distribution q(r) corresponds to a two-layer structure. At larger values of *G*, we see the deviation from the classical picture. For G=2,3, there is a plateau between the positive and negative regions. By using the local charge distribution, it is possible to obtain the integral characteristic: the cumulative charge and its dependence on the distance *r* from the micelle center Q(r), shown by

(5) $$Q\left(r\right)=\int_{0}^{r}q\left(x\right)dx=4\pi \int_{0}^{r}\left(\varphi_{term}\left(x\right)+\varphi_{Na}\left(x\right)-\varphi_{Cl}\left(x\right)\right)\cdot x^{2}dx$$

In Figure 8c, a single characteristic maximum of Q(r) for G=1 is observed. The maximum Q(r) for G=2–3 is not so distinct. According to the location of the maxima, the distribution of the number of the terminal charged groups $n_{t}\left(r\right)$ (Figure 8d) is similar to q(r).

**Figure 9:**
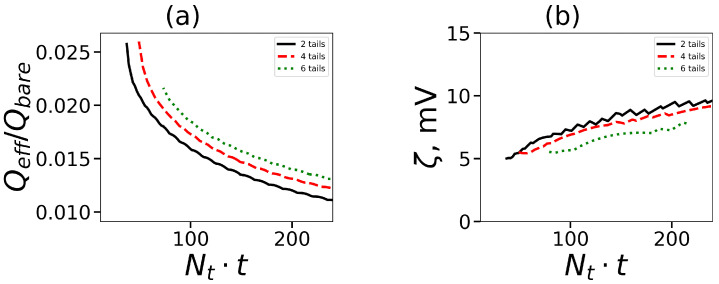
(**a**) The relative effective charge and (**b**) the zeta-potential ζ (in Volt) at different amounts of hydrophobic segments in tails. All cases correspond to the same generation number G=4.

The dependency Q(r) has just one maximum for typical macroions. The location of the right maximum can be considered as an effective radius $R_{eff}$ of a macroion. This value was also used earlier in Figure 5 and marks the boundary between the interior of the micelle and the diffusion cloud of counterions outside. The effective charge of the micelle $Q_{eff}$ is the value of the shaped charge at a distance equal to $R_{eff}$. We define the relative effective charge $Q_{eff}/Q_{bare}$ where $Q_{bare}$ equals the number of terminal charged groups $N_{term}$. Its dependence on $N_{t}\cdot t$ is shown in Figure 9a. The effective charge decreases with $N_{t}\cdot t$. It means that the larger number of counterions neutralize charged groups of lysine dendrons. The increase in the number of tails (for the given amount of hydrophobic segments in the surfactant) leads to an increase in the effective charge.

Another critical characteristic of macroions is a zeta potential ζ which is equal to the value of the electrostatic potential ψ(r) at the $R_{eff}$ point. Zeta potential shows the behavior opposite to that of the effective charge (see Figure 9b). It increases with an increase in $N_{t}\cdot t$, and decreases with the growth of the number of tails for the given overall number of hydrophobic segments in the surfactant.

### 2.4. The Stratifications in Dendrons

**Figure 10:**
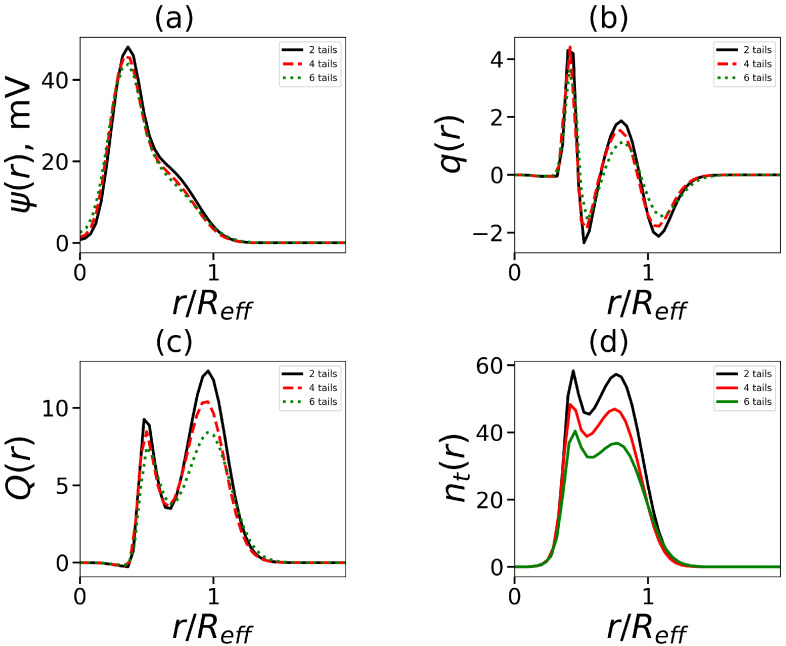
The normalized radial distribution (by effective radius $R_{eff}$) (**a**) the electrostatic potential ψ, (**b**) the dimensionless charge q(r), (**c**) the cumalative charge Q(r) and (**d**) the terminal charged groups of dendrons in the micelle. All cases correspond to the same overall number of hydrophobic segments in the surfactant $N_{t}\cdot t=120$ (t=2, $N_{t}=60$; t=4, $N_{t}=30$; t=6, $N_{t}=20$) and generation number G=4.

In this part, we will focus on molecules with dendrons G=4 (this is the threshold value, up to which generations of dendrons can be conditionally referred to as small and beyond that—large; usually, the density in dendrons begins to grow from this generation [66]). In contrast to small generations of dendrons, which only have one decay zone (see Figure 10a), the electrostatic potential has two decay zones (see Figure 8a). The charge number distribution has two non-standard maxima and negative regions: an inner region where counterions entering the micelles corona are localized, and an outer region (see Figure 10b). This situation is also seen in the cumulative charge, which now has two maxima (see Figure 10c). This is distinct from the standard double-layer pattern (see Figure 8b,c). The aggregation number falls as the number of tails rises, which in turn results in fewer dendrons in the corona and a lower seed charge. This affects the reduction in the second peak of the charge Q(r). Additionally, it causes the number of end groups in the corona to decline (this can be seen in Figure 10). Two local maxima in Figure 10d coincide with equivalent maxima in the charge distribution.

The stratification effect was shown recently for planar brushes formed by short and long linear chains or star-like polyelectrolytes [67,68]. For such mixed brushes, the theory predicts the internal segregation of tethered chains into weakly and strongly extended populations by considering the finite extensibility of chains. As a result, an internal double electrical layer is created, with the narrow cloud of counterions localized at the interface between the layers inside a stratified brush.

In our case, there are also two maximums. What is the reason for the stratification that has appeared in our case? Two assumptions can be put forward: (1) the stratification arises inside each dendron: due to the asymmetry of lysine dendrons, the terminal groups of branches with small contour lengths are compressed and located in the first population, while the terminal groups of branches with longer contour lengths are elongated and form the second population; and (2) the dendrons, as a whole, split into two populations, one of which is entirely located close to the core, and the other which is elongated towards the periphery.

**Figure 11:**
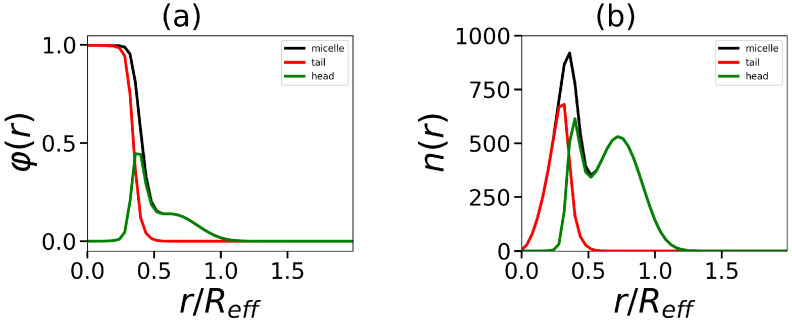
The radial distributions of segments in the hydrophobic core and hydrophilic corona, as well as the total distribution: (**a**) the volume fraction φ(r) and (**b**) the number distribution n(r). All cases correspond to the same number of tails t=2, tail length $N_{t}=60$, and generation number G=4.

**Figure 12:**
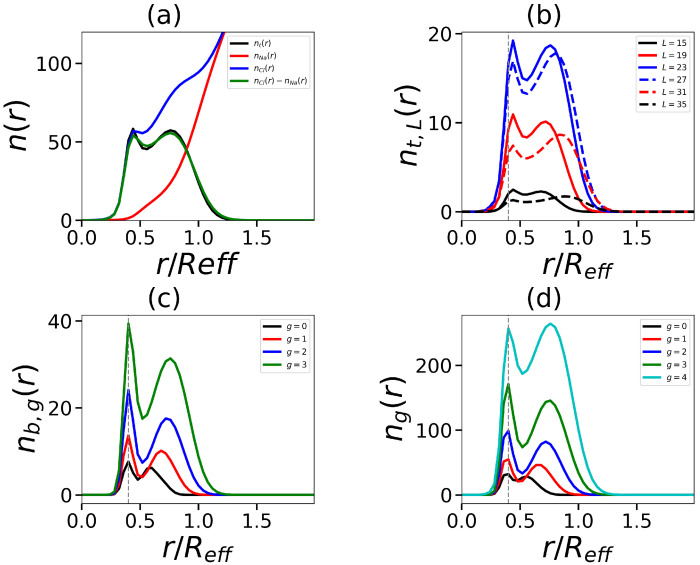
The radial distribution of (**a**) the terminal charged groups of dendrons, co-ions, and counterions, (**b**) the terminal charged groups with different contour lengths, (**c**) the branch points from different subgenerations, and (**d**) the dendron segments from different subgenerations. All cases correspond to the same number of tails t=2, tail length $N_{t}=60$, and generation number G=4. The vertically dotted gray lines on the graphs (**b**–**d**) indicate the location of the core size $R_{core}$.

We have tested these assumptions on a model with two tails (Figure 11). For corona segments, we see the density profile φ(r) with two different slopes (Figure 11a) and the division of the number segment distribution n(r) into two populations (Figure 11b). The detailed analysis shows that in addition to the stratification of the distribution of all terminal groups $n_{t}\left(r\right)$ (Figure 12a), one also observes the stratification into two populations for terminal groups with different contour lengths $n_{t,L}\left(r\right)$ regardless of their contour length (Figure 12b). The same is valid for branch points for various sub-generations (Figure 12c), and the total number of segments across sub-generations (Figure 12d).

Therefore, the second assumption happens to be valid: dendrons, as a whole, choose which of two populations they want to be in. The first population is located near the core, and the second is close to the periphery. Counterions fill the “gap” (in terms of charge distribution, as the overall density is constant in this intermediate region) between the two populations. Such separation occurs since hydrophilic dendrons do not overlap well [69]. We also note that the first population of dendrons is severely pressed to the core (Figure 12). These pressed dendrons of the first population are found at the foot of the elongated dendrons of the second population, which stretch towards the periphery of the corona, trying to occupy the free space for their branches.

## 3. Materials and Method

**Figure 13:**
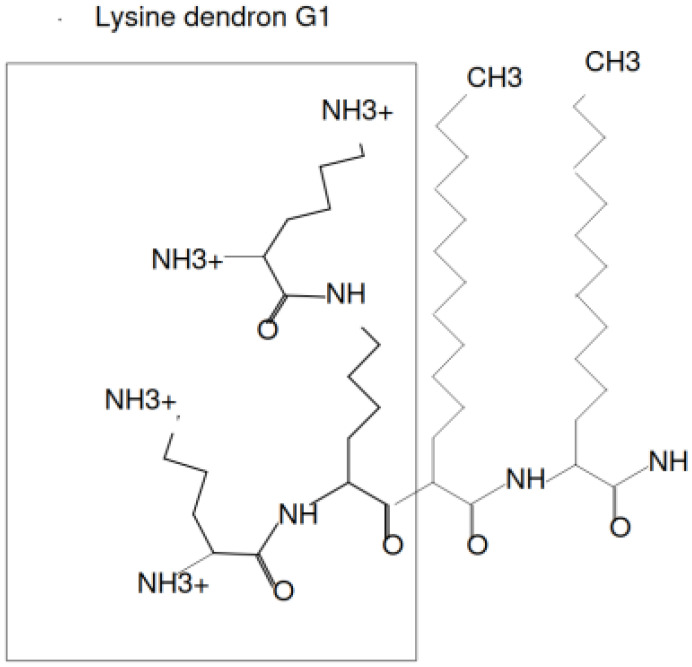
The chemical structure of the hybrid molecule (surfactant) consisting of a lysine dendron G=1 with two hydrophobic tails t=2. The number of charged groups NH${}_{3}^{+}$ equals $2^{G+1}=4$. The number of hydrophobic segments CH${}_{2}$ in a tail is $N_{t}=12$.

In this paper, we study the chemical structures similar to that presented on Figure 13. The structure contains a lysine dendron and several hydrophobic tails. The dendrimer molecules were named in literature as “unimolecular micelles”, even without additional hydrophobic tails [70,71] because they usually have relatively hydrophobic interior and hydrophilic terminal groups. Some theoretical (mean-field, self-consistent field, as well as molecular, Brownian, and stochastic dynamics simulation) approaches were applied to study different dendrimers [69,72,73,74,75,76,77]. However, dendrimers of high generations are toxic due to many of their positively charged terminal groups. To avoid the problem, it is possible to use dendrimers of lower generations [78,79,80,81], or modify the terminal groups of dendrimers by noncharged or negatively charged groups, for example, hydroxyl or carboxyl [82]; or by functional groups [83]; or link terminal groups to drug molecules [84]; or move at least parts of the charges from terminal groups to internal spacers of the dendrimer [85,86,87]; or link terminal groups to linear hydrophilic tails, for example polyethylene glycol (PEG) [88,89]. It is also possible to design hybrid amphiphilic molecules consisting of one dendron with one or more linear hydrophobic tails attached to its root (Figure 13) [9,90,91,92].

**Figure 14:**
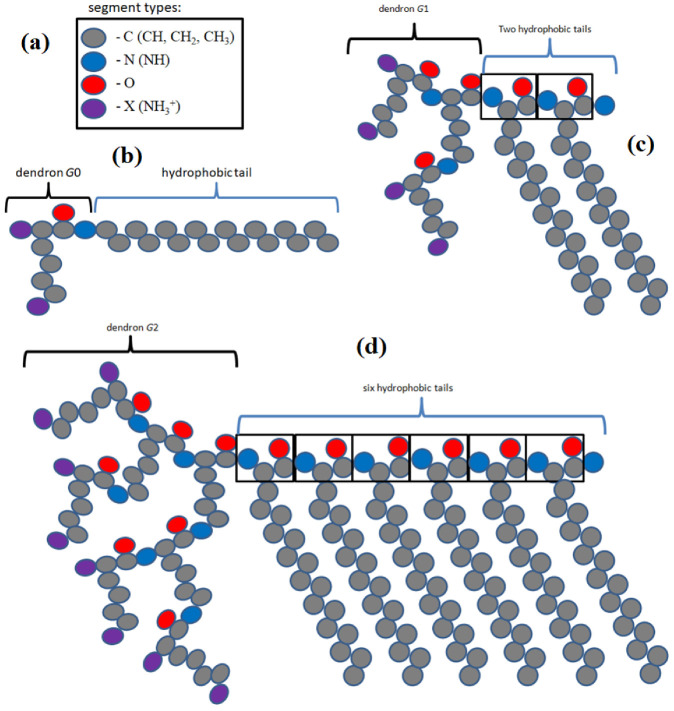
The united atom model: (**a**) segment types in hybrid molecules, (**b**) the model with single chain (t=1) and G=0 [9], (**c**) the model with two chains (t=2) and G=1 [10], and (**d**) the model with six chains (t=6) and G=2 [10]. Each tail on this graph consists of 16 CH${}_{2}$, i.e., $N_{t}=16$.

The self-assembly of such hybrid molecules composed of lysine dendron with the generation number *G* and a hydrophobic part was considered in this paper. The hydrophobic part may contain several (from 1 to 6) ${\left(CH_{2}\right)}_{n}$ tails ($N_{t}$ is the number of $CH_{2}$ groups in the tail). For brevity, we will call such molecules surfactants. As an illustration, Figure 13 shows the chemical structure of a hybrid molecule with a lysine dendritic head (G=1) and two hydrophobic tails (t=2). These molecules are immersed in water containing salt ions. In this paper, the following conditions were chosen: the salt concentration is equal to 0.1 M (physiological conditions), and the system is at room temperature. In the calculations, the atomistic model of molecules (Figure 13) is replaced by a model of united atoms (Figure 14). This model considers explicitly heavy atoms, and combines light hydrogen atoms into a group with the corresponding heavy atoms. The experimental work [10] was chosen as a basis for constructing the model of one-, two-, or more-tailed hybrid molecules. A similar approach was used in our previous work [65] to construct hybrid molecules with one tail [9].

The Flory interaction parameters (see Table 1) for our model provide a good agreement between numerical calculations and experiments for micelles of hybrid molecules with one tail [65]. These parameters were chosen for the first time in [93], where they were successfully applied to simulate lipid membranes. The driving force of self-assembly is the hydrophobicity of the tails. Therefore, the repulsion parameter Flory between water and carbon $\chi_{C,W}=1.2$ [94] became the critical parameter. It is known that the core of micelles is obtained dry, (without water) and counterions, and the heads of hybrid molecules do not penetrate into it. This explains the choice of repulsive Flory parameters for the interactions of carbon with counterions $\chi_{C,Na}=\chi_{C,Cl}$ and with the soluble parts of the heads $\chi_{C,NH}=\chi_{C,O}=\chi_{C,NH_{3}^{+}}=2$. Because NH and O form hydrogen bonds with water and are hence hydrophilic, repulsive Flory parameters $\chi_{W,NH}=\chi_{W,O}=-0.6$ were chosen. For a more detailed description of the parameter selection, see the works [65,93].

The segment valencies $\nu_{X}$ are specified in Table 1. Amino groups $NH_{3}^{+}$ of lysine are fully charged, i.e., we assume low pH conditions. Table 1 also contains the relative dielectric permittivities. For the hydrocarbon phase for the bulk water, respectively, the dielectric permittivities between the two limiting cases are 2 and 80 [65].

The lattice size *b* is used to introduce the characteristic lengths in electrostatic interactions. We used the value $b=3\times 10^{-10}$ m. The numerical method uses dimensionless lengths and densities. For conversion to physical dimensions, special multipliers are used. The cell size *b* is used to translate lengths, i.e., [size in nm] = b× [dimensionless size]. The formula for converting from dimensionless density: [concentration in M] = k× [dimensionless density φ], where k=10 (for the calculation and choice of the parameter *k*, see our previous work [65]). For example, the dimensionless volume fraction of salt $\varphi_{Na}^{b}=0.01$ corresponds to a salt concentration of 0.1 M. For details on the use of the numerical method for the self-assembly of molecules of this model, see the work [65]. Further details of computer programs used in simulations and for calculation of different characteristics of studied systems could be found elsewhere [95,96,97,98,99,100].

## 4. Conclusions

In this paper, the self-assembly of amphiphilic hybrid molecules in water, consisting of one lysine dendron and several hydrophobic tails attached to its root, was studied using the numerical self-consistent field approach. All calculations were performed at the salt concentration 0.1 M corresponding to physiological conditions. Primary attention was paid to spherical micelles as the most convenient shape of carrier candidates for drug and gene delivery into cells. The diagrams of the states of the micelles in coordinates of the overall number of segments in hydrophobic tails and the generation number *G* in the hydrophilic dendron were determined. The main conclusion of this part is that a hybrid molecule with a small number of long hydrophobic tails must have a larger dendritic head to form a micelle than a molecule with a large number of short tails containing the same total amount of hydrophobic monomers.

For spherical micelles, the relationship between the structure of hybrid molecules and resulting aggregates was studied. It was shown that the spherical micelles have a dense core containing hydrophobic monomers of tail and a loose corona containing monomers of lysine dendrons, counterions, and salt ions. For the same generation number *G* of the dendron and tail number *t*, the aggregation number of the micelle grows monotonously with the growth of the tail length. The thickness of the corona decreases both, with an increase in the total number of hydrophobic segments, and with an increase in the number of tails, provided that the total number of segments in the tails remains unchanged. One of the main results is that the splitting of hydrophobic segments over a larger number of tails leads to an increase in the core density and a decrease in both the aggregation number and the number of terminal charged groups in the micelles.

Special attention was paid to the electrostatic properties of the resulting micelles. It was shown that due to the penetration of counterions into the dendron corona, the relative effective (uncompensated) charge of the micelle decreases, and the zeta-potential increases with an increase in the total number of hydrophobic segments in the surfactant tails. On the other hand, an increase in the number of tails (at constant total number of hydrophobic monomers) leads to an increase in relative effective charge, and a decrease in zeta-potential. It is interesting that the charge distribution in the corona depends on the generation number *G* of a dendron in the surfactant. At the small number of generations G≤3, a standard double electrical layer is observed. At the larger number of generation (G=4), a stratification of dendrons in the corona is observed. Dendrons in the shell are divided into two populations: first population is close to the spherical core, and the second population is close to the spherical surface of the micelle. As a result, the charges of dendrons are divided into two populations also. Since the width of the intermediate region is quite large, the bioactive molecules can penetrate into this region. The last result is of potential interest for the use of studied spherical dendromicelles as nanocontainers for drug delivery.

## Figures and Tables

**Table 1:** (**a**): a set of Flory-Huggins interaction parameters χ between two distinct segments. The segment types W (water), C (CH, CH${}_{2}$, CH${}_{3}$), charged units are surfactant NH${}_{3}^{+}$, neutral NH and O, the generic co-ion Na${}^{+}$ and counterion Cl${}^{-}$. (**b**): valency $v_{X}$ of the segments *X*, and relative dielectric permittivity $\varepsilon_{rX}$.

(**a**)							
χ	W	C	NH${}_{3}^{+}$	NH	O	Na	Cl
W	0	1.2	0	−0.6	−0.6	0	0
C	1.2	0	3	2	2	2	2
NH${}_{3}^{+}$	0	3	0	0	0	0	0
NH	−0.6	2	0	0	0	0	0
O	−0.6	2	0	0	0	0	0
Na	0	2	0	0	0	0	0
Cl	0	2	0	0	0	0	0
(**b**)							
*v*	0	0	1	0	0	1	−1
$\varepsilon_{r}$	80	2	5	5	5	10	10

## Data Availability

Not applicable.
